# Analysis of the intrinsic value of life in the context of synthetic biology

**DOI:** 10.3389/fbioe.2025.1536403

**Published:** 2025-03-31

**Authors:** Yi Zhang, Yuling Chen, Bohua Liao

**Affiliations:** ^1^ Office of Academic Research, Fujian Institute of Socialism, Fuzhou, China; ^2^ School of Marxism, Ningbo University of Finance and Economics, Ningbo, China; ^3^ College of Rural Revitalization, Fujian Agriculture and Forestry University, Fuzhou, China

**Keywords:** synthetic biology, synthetic life, value, intrinsic value, moral status

## Abstract

The ongoing advancements in synthetic biology, employing either “bottom-up” or “top-down” approaches to construct synthetic life, are generating significant interest. However, the broad application of these scientific practices remains fraught with ethical controversies. Thus, investigating the intrinsic value associated with synthetic life is crucial for determining whether and how synthetic life should be constructed and utilized. This study draws upon and extends Ronald Sandler’s theory of intrinsic value, analyzing the intrinsic subjective value of synthetic life from the perspectives of ecocentrism, human culture, and the structural properties of synthetic life itself. It examines the intrinsic objective value of synthetic life based on its natural purposes. Additionally, the study explores the inherent worth of synthetic life from three angles: biology, subjectivity, and relationships with human beings. We conclude that the intrinsic value of synthetic life increases sequentially from synthetic microorganisms to synthetic plants, synthetic invertebrates, synthetic vertebrates, and synthetic humans. All forms of synthetic life possess intrinsic subjective and objective value. However, only synthetic life above the grade of synthetic microorganisms has inherent worth; thus, humans have moral obligations towards them.

## 1 Introduction

Synthetic biology is an interdisciplinary field that integrates multiple disciplines. Although there is not yet a unified definition of synthetic biology, it is clear that one of its ultimate visions is to achieve the “bottom-up” synthesis of “artificial cells” or “protocells” that possess fundamental life functions such as metabolism, growth, development, and adaptation (Kretschmer and Schwille, 2014). In simpler terms, it aims to achieve the *de novo* synthesis of “life.” The term “protocell” was initially used by scholars studying the origin and evolution of life. It refers to a compartment that allows primitive genetic material and catalytic products to replicate and accumulate independently of the external environment (Joyce and Szostak, 2018). Synthetic biologists attempt to assemble and construct similar cells through artificial synthesis, hoping to gain a deeper understanding of life through the “build to understand” approach (Wang et al., 2016). This “bottom-up” attempt to synthesize life challenges the traditional reductionist paradigm of biology, bestowing life and biology with new possibilities.

Specifically, the “bottom-up” practice of synthesizing life can be divided into three stages: the design and synthesis of genetic circuits, the design and synthesis of whole genomes, and the design and synthesis of whole organisms (Xiao et al., 2015). Current technological capabilities already allow for the design and synthesis of genetic circuits and whole genomes. Still, the design and synthesis of organisms cannot yet be achieved with existing techniques. Scientists first achieved the artificial synthesis of the poliovirus genome around 2000 (Cello et al., 2002), sparking great enthusiasm among virologists. In 2003, Hamilton Smith and colleagues synthesized the genome of bacteriophage φX174 (Smith et al., 2003), and Boyd Yount et al. successfully synthesized the whole genome sequence of the *Severe Acute Respiratory Syndrome Coronavirus* (SARS-CoV) using reverse transcription polymerase chain reaction (RT-PCR) technology (Yount et al., 2003). In 2005, an international research team led by American scientists discovered the eight gene segments of the 1918 influenza virus in patient tissue samples and reverse-engineered a similar 1918 influenza virus genome based on these gene segments (Tumpey et al., 2005). In 2010, a typical example of whole genome design and synthesis is “Synthia” (Gibson et al., 2010), which was created by Crag Venter and his colleagues, and more recently, synthetic biologists have achieved partial artificial synthesis of the eukaryotic yeast chromosome genome (Zhao et al., 2023).

Beyond the “bottom-up” *de novo* synthesis of life, synthetic biologists have also attempted the “top-down” deep modification of existing life forms in nature. At first glance, the “life” modified in this approach might seem no different from traditional genetically modified organisms (GMOs); however, this is not the case. There are significant quantitative and qualitative differences between the two.

Quantitatively, traditional genetic modification techniques can only transfer a few genes between different species, whereas synthetic biology enables the transfer of large-scale gene clusters and even the reconstruction of entire metabolic pathways. A typical example is bioengineered microorganisms. These are the integration of genetic engineering and synthetic biology, where genetic engineering can modify the genetic code of target microorganisms and introduce various genes from others, and synthetic biology can synthesize or even create new genes and further reconstruct intracellular metabolic pathways. Together, these approaches can enable bioengineered microorganisms to achieve desired traits or functions (Tucker and Zilinskas, 2006). In this field, bioengineered microorganisms can be classified into prokaryotic microorganisms, represented by *Escherichia coli*, and eukaryotic microorganisms, represented by yeast. György Pósfai and colleagues used synthetic biotechnologies to reduce the genome of *Escherichia coli* by 15% while maintaining reasonable growth characteristics (Pósfai et al., 2006). Marc J. Lajoie et al. replaced all known UAG stop codons in *Escherichia coli* with synonymous UAA codons, endowing it with antiviral capabilities (Lajoie et al., 2013). In 2006, Jay Keasling and scientists at the University of California, Berkeley, utilized synthetic biotechnologies to produce the low-cost fermentation of artemisinin precursors in yeast (Ro et al., 2006). In 2014, the pharmaceutical giant Sanofi further transformed the artemisinin precursors produced by yeast fermentation into artemisinin, thereby realizing the practical application of biopharmaceuticals (Peplow, 2016). In 2015, researchers at Stanford University introduced a series of engineered bio-modules into eukaryotic yeast, enabling yeast to ferment sugars to produce opioid compounds. This method can avoid yield instability caused by external factors (such as climate change and pests), making drug production more efficient and convenient (Galanie et al., 2015).

Qualitatively, both traditional genetic modification techniques and selective breeding require that the “genes” and “traits” must already exist in nature. These techniques cannot create genes or traits that do not exist in nature. Synthetic biology, however, can develop entirely new genes and traits that do not exist in nature. Examples include xenobiology and biorobotics. Xenobiology is a branch of synthetic biology that focuses on constructing xenonucleic acids (XNA), expanding genetic codons, and developing novel polymerases (Schmidt et al., 2018). Biorobotics is an emerging field that studies the combination of living cells with mechanical engineering devices, mainly focusing on biohybrid robots, which are the products of the integration of synthetic biology and mechanical engineering (Ayers, 2018).

Scientifically, both “top-down” and “bottom-up” synthesis of life using synthetic biotechnologies are indisputable facts. Philosophically, beyond general scientific philosophical questions, the value of life in the context of synthetic biology has sparked broader and more intense debates. For example, from a biocentric perspective, Robin Attfield argues that synthetic life has a certain moral status and intrinsic value, but different types of life have different moral statuses (Attfield, 2012). Bernard Baertschi, however, believes that the origin of life is irrelevant to its moral status. Even if life is created by humans using modern biotechnologies rather than by nature, its moral status or intrinsic value remains unchanged (Baertschi, 2012). Lewis Coyne argues that synthetic life appears because of its extrinsic value but possesses intrinsic value as inherent to life, distinguishing it from machines that only have extrinsic or instrumental value (Coyne, 2020). One of the main tasks of this paper is to address the value controversy of synthetic life. Additionally, we will answer a related and more pressing question: How should humans treat or even utilize synthetic life?

Before that, we must define “synthetic life,” as discussed in this paper. As is well known, defining life is a challenging problem, and there is even more controversy over the definition of “synthetic life.” Traditionally, artificial life has three dimensions: “software,” which simulates or creates life-like behaviors using computer programs; “hardware,” which uses mechanical devices to study the creation of life-like systems; and “wet,” which uses biochemical materials in the laboratory to create life-like systems (Bedau et al., 2010). According to this classification, “synthetic life” clearly belongs to the “wet” category. However, with the development of silico synthetic biology (Konur et al., 2021) and biorobotics, there is a trend to integrate the three dimensions into “synthetic life,” making the definition even more complex.

Given that this paper’s objective is not to provide a precise definition of “synthetic life,” to clarify the subject of this discussion and the related arguments, we offer a rough definition of synthetic life: (a) Life created according to human intentions or purposes through the chemical synthesis of non-living materials found in nature; (b) Life that, according to human intentions or purposes, is deeply modified using (synthetic) biotechnologies to possess new traits or functions that natural life does not have. We believe this definition encompasses both the “bottom-up” synthesized life or life-like entities in synthetic biology (even though it may not be currently achievable) and the “top-down” deeply modified natural life, enabling a more comprehensive discussion of artificial life in the context of synthetic biology.

After clarifying the subject of this discussion, we further need to clarify the core issue discussed in this paper, namely the intrinsic value of synthetic life. Meanwhile, we will explore the connotation of synthetic life’s intrinsic value to provide a moral foundation for how humans should treat them.

## 2 The axiological issues of synthetic life

In axiology, the value of something can be divided into “intrinsic value” and “extrinsic value”(Hart, 1971). Discussions about intrinsic value and extrinsic value (instrumental value) often involve the relationship between “ends” and “means.” Ends are often referred to as “intrinsic value” because they are contained within something itself; means are referred to as “extrinsic value” or “instrumental value” because their value is derived from their usefulness in achieving or maintaining the intrinsic value of something (Bahm, 1993). Philosophers generally consider intrinsic value more important because of its inherent, non-derivative nature, and they believe that extrinsic value should be explained by intrinsic value (Yee, 2015). However, this is the relationship between intrinsic and extrinsic value in a general sense and does not necessarily apply to the intrinsic and extrinsic value of a specific thing. For example, a tree has intrinsic value in maintaining its own growth and development and achieving its life purposes. At the same time, it also has an extrinsic value derived from providing raw materials for building houses and meeting human needs (which are the intrinsic value of human lives) for shelter and warmth. Generally (although some disagree), people believe that sacrificing the intrinsic value of trees to meet basic human needs can be ethically justified. However, when similar situations involve vertebrates or even another person, the scenario changes significantly, as it involves weighing different intrinsic and extrinsic values. Therefore, we should first examine whether a specific thing has intrinsic value and then consider its extrinsic value when conducting value analysis and ethical weighing. This means we should first determine its natural purposes, moral status, moral rights, etc., and on this basis, determine whether and how it can be used as a means/tool and what moral considerations should be in place when viewing it as a means/tool.

The significant difference between synthetic biology and traditional science and technology lies in its capacity to actively involve humans in creating life for the first time. Therefore, the issue of the value of synthetic life is at the core of research on the axiology of synthetic biology and its technologies. On the one hand, it reflects the unique intrinsic value of these distinct “creations,” and on the other hand, it reflects their great potential extrinsic value to humanity. The substantial extrinsic value of synthetic life can easily lead humans to neglect its intrinsic value and manipulate it arbitrarily. Thus, before developing and utilizing these new species, we should focus on analyzing their intrinsic value. Through this analysis, we can determine our moral responsibilities towards different categories of synthetic life, the degree of development, protection, and utilization of their extrinsic value, and what harm we should avoid causing them. Specifically, this involves the moral status of synthetic life. Moral status means that when an entity has moral status, moral agents owe or may owe moral obligations to it. Moral agents must consider the entity’s interests to some extent and cannot arbitrarily dominate it (Sher, 2017). It is generally believed that different types of life have different intrinsic values and, therefore, different moral statuses (for example, organisms capable of experiencing pain have higher moral status than those incapable of experiencing pain). Humans have the highest moral status, so when human interests conflict with other lives, sacrificing other lives to protect human interests can be justified to some extent. However, this sacrifice is limited, as humans, as moral agents, must consider the basic interests of other life with moral status.

For synthetic life, which is originally a natural entity but created through artificial synthesis, there is controversy over how to recognize and evaluate its intrinsic value and assign it corresponding moral status. Notably, many scientists view synthetic life as a machine or at least a special “living machine” (Ebrahimkhani and Levin, 2021). They consider natural life as “imperfect machines” shaped by natural selection, and a major task of synthetic biology is to improve and repair these “imperfect machines” to make them “perfect machines” (Morange, 2012). Such controversies not only impact whether we can create and utilize synthetic life but also determine to what extent we can utilize synthetic life (sacrificing its interests) and whether the dignity of life is violated.

So, does synthetic life have intrinsic value? If it does, how should we treat it (i.e., what is its moral status)? In discussing the intrinsic value of synthetic life, we draw on Ronald Sandler’s theory, which divides intrinsic value into three aspects: intrinsic subjective value, intrinsic objective value, and inherent worth (Sandler, 2012). The reason for adopting Sandler’s theory is that it can cover a wide range of life forms, from low-grade to high-grade synthetic life. In the traditional deontological framework, it is difficult to include lower-grade life forms that lack personhood characteristics, while utilitarianism focuses more on things external to life itself.

It is important to note that we are not the first to introduce Sandler’s theory of intrinsic value into the discussion of the value of synthetic life. Previously, prominent artificial life researchers Mark A. Bedau and Ben T. Larson explored synthetic life’s intrinsic value from the perspective of environmental ethics using Sandler’s theory. They concluded that (i) Due to the subjective nature of intrinsic subjective value, all forms of synthetic life have intrinsic subjective value; (ii) the birth of synthetic life often involves rational design and directed evolution in experiments, and these entities themselves also possess the ability for random mutation, giving them a “history” similar to natural evolution, thus conferring intrinsic objective value; (iii) like natural life, synthetic life is a living entity with its own interests and needs, and therefore also has inherent worth (Bedau and Larson, 2013). We partially agree with this conclusion, acknowledging the necessity and persuasiveness of introducing Sandler’s theory into discussing the value of synthetic life. However, we also believe that Bedau and Larson’s discussion was overly simplistic, and a more in-depth exploration of related issues is needed.

First, is the intrinsic value of different types of synthetic life homogeneous? If not, what are the criteria for evaluation and differentiation? They did not address this in the original text. Second, intrinsic subjective value, intrinsic objective value, and inherent worth all require more detailed exploration from different dimensions. The discussion of inherent worth is particularly insufficient because the inherent worth of synthetic life involves human moral obligations and determines whether and how humans can use it, which in turn affects whether the dignity of life as a whole is violated. The original discussion attributed inherent worth to biological interests and needs, which is insufficient and even incorrect. Our analysis reveals that some synthetic life, such as bioengineered microorganisms, have biological “goods,” but they do not have inherent worth. Third, although current synthetic biology focuses on simple life forms, we should not limit its vision to existing matters. It should adopt a forward-looking and precautionary approach to potential future developments in this field, especially the significant impact of synthesizing higher-grade life forms (such as vertebrates). Therefore, the study of the intrinsic value of synthetic life must analyze all types of life forms. This paper refines Sandler’s intrinsic value theory, analyzing the intrinsic subjective value of synthetic life from the perspectives of ecocentrism, human culture, and the structure of synthetic life itself. It analyzes the intrinsic objective value of synthetic life based on its natural purposes and the inherent worth of synthetic life from three perspectives: biology, subjectivity, and its relationship with humans. After that, we also create a hierarchy of the moral status of different types of synthetic life.

## 3 The intrinsic subjective value of synthetic life

Intrinsic subjective value refers to the type of value that something possesses because it is valued for its own sake rather than its utility (Sandler, 2012). The uniqueness of intrinsic subjective value lies in the fact that it exists because it is a value generated by someone’s evaluative attitude, which has the right relationship with someone’s subjective acts of attributing intrinsic value (Bedau and Larson, 2013). People may attribute different intrinsic subjective values to certain things due to their content, historical or socio-cultural significance, or even scarcity. From this perspective, does synthetic life possess such intrinsic subjective value?

Firstly, for some people (especially ecocentrists), nature is valued for its sanctity, mystery, and uniqueness and thus should be revered. From this viewpoint, any form of life is part of nature and should be respected. Synthetic life, being a form of life and part of nature, should be considered sacred and unique to humanity, thereby possessing intrinsic subjective value. However, different types and grades of (synthetic) life possess different degrees of intrinsic subjective value. Humans undoubtedly attribute different levels of importance or reverence to synthetic microorganisms, synthetic plants, synthetic invertebrate, synthetic vertebrate, and synthetic humans (again, which may be ethically prohibited), leading to varying degrees of intrinsic subjective value. Just as people instinctively consider saving an elephant more commendable than saving a bacterium, the same applies to synthetic life. Therefore, from a moral intuition perspective, we can infer that synthetic humans (if possible) would have greater intrinsic subjective value than typical synthetic vertebrates. But how should we rank the intrinsic subjective value of synthetic vertebrates, invertebrates, plants, and microorganisms? This requires new variables and perspectives.

Secondly, from a cultural perspective, synthetic biology represents scientific and technological progress, embodying the crystallization of human wisdom and a symbol of culture. The synthetic life created through synthetic biotechnologies concretely represents this crystallization and symbol, carrying significant socio-cultural and historical meaning. Specifically, the scientific practice of “bottom-up” attempts at *de novo* synthesis of life marks a shift in biology from a “descriptive” to a “possible” science (Ijäs and Koskinen, 2021). This “building to understanding” signifies a new epistemological approach to approaching “truth,” and synthetic life is also a testament to this “epistemological revolution.” Therefore, from this perspective, synthetic life also possesses intrinsic subjective value. Similarly, the cultural, social, and historical significance of different types of synthetic life vary. For example, the scientific significance of synthesizing a prokaryotic *Mycoplasma* genome differs from that of synthesizing a eukaryotic yeast chromosome, with the latter undoubtedly carrying greater cultural, social, and historical value.

Thirdly, considering the structure of synthetic life itself, some people might marvel at its exquisite construction, appreciating it purely from an aesthetic perspective and considering it a “work of art.” Hence, synthetic life also possesses aesthetic value. In the field of bioart, artists can use synthetic biotechnologies to manipulate cells, tissues, and even creatures to enhance or achieve related artistic expression (Byerley and Chong, 2015). In the field of bioart, artists can use synthetic biotechnologies to manipulate cells, tissues, and even creatures to enhance or achieve related artistic expression (Byerley and Chong, 2015). For instance, the 2009 “Synthetic Aesthetics” project in Science, Technology, and Society (STS) aimed to bring together synthetic biologists and artists for interdisciplinary exchange. Based on this project’s research, artist Alexandra Daisy Ginsberg and others published the book “Synthetic Aesthetics” (Calvert and Schyfter, 2017). From this perspective, synthetic life also possesses intrinsic subjective value. Similarly, the higher-grade synthetic life, the more intricate their structures, and the greater their aesthetic significance and value.

Combining these three aspects, we conclude that synthetic humans (if possible) have the greatest intrinsic subjective value, followed by synthetic vertebrates, synthetic invertebrates, synthetic plants, and synthetic microorganisms, respectively.

However, it is crucial to note that this intrinsic subjective value is directly determined by human attitudes, which are easily influenced by the usefulness or harmfulness of synthetic life. For instance, bioengineered microorganisms producing opioid precursors on a large scale to relieve humans’ pain may be highly valued by some, while others might view them as exacerbating narcotic and drug abuse, thus valuing them less. Synthetic pathogens, perceived by scientists as promoting scientific progress and vaccine development with extraordinary value, may be seen by the general public as potentially causing new pandemics and, thus, valueless. Therefore, when it comes to specific types or individual instances of synthetic life, subjective evaluation standards are often influenced by the extrinsic value or potential harm they bring. This significant subjectivity can lead to assigning different intrinsic subjective values to the same synthetic life. Hence, it is insufficient to conclude that synthetic life has intrinsic subjective value merely; we need to further discuss the intrinsic value of synthetic life on other levels.

## 4 The intrinsic objective value of synthetic life

Intrinsic objective value refers to the value that something has in itself, regardless of whether anyone actually values it. According to this definition, intrinsic objective value is the value that something possesses due to its own attributes, and valuers can only recognize or discover this value, not create it (Sandler, 2012). In certain perspectives (especially ecocentrism), “natural” or having a natural evolutionary history is also a characteristic of this intrinsic objective value. These perspectives emphasize the “natural value” of life, arguing that a series of natural attributes in life determine its intrinsic objective value (Rolston, 1989), or consider naturalness itself as a source of intrinsic value (Elliot, 1992). From this standpoint, some people may argue that synthetic life created through synthetic biotechnologies does not equate to wild species of the same form. We believe this perspective is flawed.

On the one hand, the view that “synthetic life” is unnatural presupposes a dichotomy between “natural” and “artificial” or the existence of a predefined boundary between “natural” and “artificial.” But does such a boundary indeed exist? This is a matter of contention. For instance, J. Baird Callicott argues that this dualism resembles the outdated Cartesian “mind-body dualism,” and Darwin has long confirmed that humans are part of nature because humans, like all other living beings, are products of the evolutionary process (Callicott, 2008). Bruno Latour even contends that there is no purely human-unaffected nature, suggesting an interdependence between humans and nature: “Both are constructed thanks to a comprehensive network of symmetrical actions with no option for any agent to stay outside the relations of the network” (Latour, 1993). Bill Devall also puts forward a similar viewpoint: “The person is not above or outside of nature. The person is part of creation ongoing” (Devall, 1980). Thus, how can the synthetic life created by humans, who are part of nature, be considered unnatural?

On the other hand, even if “natural” has value, this does not mean that the “artificial” lacks value, nor does it prove that synthetic life lacks intrinsic objective value due to its lack of normal evolutionary history. This is because any life possesses natural purposes, leading it towards self-stabilization, self-maintenance, and self-preservation. It is necessary here to recall Kant’s words: “Life is the faculty of a substance to determine itself to act from an internal principle, of a finite substance to change, and of a material substance (to determine itself) to motion or rest, as change of its state” (Kant, 2004). These natural purposes of life, whether natural or synthetic, objectively exist as long as it is life, independent of human will. In other words, even if a synthetic life is artificially deprived of the ability to grow, reproduce, and propagate, it will still possess and continuously operate its natural purposes of self-stabilization, self-maintenance, and self-preservation. Even if humans 1 day achieve the synthesis of life from scratch, and each part of this life serves human purposes, as long as it is recognized as life, their parts must also interact and depend on each other as mutual ends (Nicholson, 2013), achieving the self-stabilization, self-maintenance, and self-preservation of life. Although such synthetic life comes into existence because of humans, once created, they will exist for their own sake (even if humans largely determine their mode of existence). As a metabolizing life entity, they will still exhibit purposeful behaviors—striving to survive as much as possible (Coyne, 2020). It also aligns with Sandler’s definition of intrinsic objective value: whether humans agree or not, synthetic life inherently possesses natural purposes, and possessing these purposes implies having intrinsic objective value.

Moreover, in scientific practice, the reproductive and propagation capabilities of synthetic life, as proof of intrinsic purposes, often cannot be entirely ignored or deprived. Designing or creating life does not mean treating them purely instrumentally or disrespecting their intrinsic purposes (Link, 2013). This point is also evidenced in the scientific practice of synthetic biology, where the natural purposes of synthetic life cannot be ignored. Taking “Synthia (JCVI-1.0)” as an example, after successfully creating JCVI-syn1.0, scientists considered whether they could eliminate nonessential genes from the *Mycoplasma* genome to achieve the vision of a “minimal genome,” leading to the later JCVI-syn2.0 and JCVI-syn3.0 (Hutchison et al., 2016). However, despite JCVI-syn3.0 being claimed to be capable of autonomous metabolism and reproduction with only 473 genes, its descendants exhibited morphological abnormalities. It wasn’t until 2021, when American scientist James F. Pelletier and his coworkers added seven additional genes to the JCVI-syn3.0 genome (creating JCVI-syn3A), that this issue was resolved (Pelletier et al., 2021). JCVI-syn3A is currently known as the minimal genome capable of normal growth and reproduction. JCVI-syn3A emphasizes “normal” self-growth and self-reproduction, with “normal” and “abnormal” being relative to natural life, meaning whether life can faithfully fulfill its natural purposes. From JCVI-syn1.0 to JCVI-syn3A, it is evident that scientists cannot entirely overlook an organism’s reproductive ability when constructing synthetic life. This self-growth and self-reproduction, for its own sake, is the best manifestation of life’s natural purposes and the best evidence of synthetic life’s intrinsic objective value.

It should be noted that in this sense, plants and microorganisms (especially pathogenic microorganisms) also possess intrinsic objective value, as they also have their own survival needs. However, this does not mean that this intrinsic objective value is related to human moral attitudes and behaviors. Simply assuming natural purposes have moral implications seems to be “question-begging” (Link, 2013). Just as the natural order dictates that carnivores eat herbivores, and herbivores eat plants, humans cannot refrain from harvesting crops due to their natural purposes of growth, nor can they avoid eliminating pathogens due to their natural purposes of survival. This intrinsic objective value cannot prevent humans from developing and utilizing them, at most imposing certain limitations—not wasting or using excessively extreme means. This ultimately appeals to ecological consequences or virtue rather than the pure intrinsic objective value of (synthetic) life.

If simply having biological needs confers intrinsic objective value, then life with rational agency and subjective experiences (perception, consciousness, beliefs, desires, emotions, etc.) would possess even greater intrinsic objective value. As such, synthetic microorganisms, synthetic plants, and synthetic invertebrates with only basic survival needs, synthetic vertebrates with pain perception abilities, and synthetic humans with emotions and rationality all possess intrinsic objective value in increasing order. However, as we have noted, merely possessing intrinsic objective value cannot determine how we ought to treat them, nor can it determine their moral status. Therefore, we must delve deeper into another type of intrinsic value of synthetic life—their inherent worth.

## 5 The inherent worth of synthetic life

Inherent worth refers to the value that something possesses due to the benefits (or interests) it inherently has, which other valuers (or moral agents) ought to care about. This inherent worth focuses on the wellbeing or flourishing of life, which grants something that owns it the right to make claims on other moral agents based on these benefits and interests (Owe et al., 2022). Thus, inherent worth involves two important elements: first, something has its own benefits or interests; second, other moral agents should care about these benefits or interests (Sandler, 2012). The basis of inherent worth partially overlaps with the basis of intrinsic objective value. For the former, more emphasis is placed on the moral obligations of humans to show concern and respect for entities with intrinsic value, or these can be regarded as the moral rights of these entities. It grants these entities a certain degree of moral status or moral consideration relative to humans, with rights to avoid harm and maintain wellbeing. A significant distinction between inherent worth and other intrinsic values is that inherent worth involves morality, particularly moral obligations. The capacity for rational reflection is the hallmark of moral action, a capability unique to humans; therefore, only humans have the capacity for moral action. Moral obligations, as normative requirements for moral actions taken by humans, are exclusively possessed by humans. Consequently, the assignment of moral status is necessarily anthropocentric. Meanwhile, when considering inherent worth, the involvement of moral obligations inherently makes this value anthropocentric.

In terms of distinguishing between different levels of inherent worth, we believe this is directly related to the degree of moral status of the entity. Different types of life possess varying levels of interests and wellbeing that humans should care about, resulting in differing levels of moral status. This point was also insufficiently addressed in the paper by Bedau and Larson. Regarding the moral status of synthetic life, we adopt a scalar conception of moral status (Degrazia, 2008), where different species are assigned different levels of moral status based on their typical characteristics. However, we believe that although moral status is divided into various levels, these levels are not further subdividable within the same category. Each entity should be treated as equal members of their respective categories (Timmer, 2023).

In this section, we will further assess and measure the inherent worth of synthetic life from biology, subjectivity, and relationships with human beings, considering the moral considerations that humans should extend to.

### 5.1 Inherent worth from the perspective of biology

All life forms have benefits, interests, and wellbeing, including synthetic life. We can also understand this from Aristotle’s teleological perspective, which emphasizes the right of life to fulfill their purposes (Coyne, 2020). These natural purposes determine their intrinsic objective value, which typically, to some extent, confers moral status (Deplazes-Zemp, 2012), and thus inherent worth. However, possessing intrinsic objective value is only a necessary condition, not a sufficient one, for having moral status. Not all life with intrinsic objective value have moral status. Lower-grade synthetic life (particularly synthetic microorganisms), while possessing intrinsic objective value and capable of self-adjustment for survival, self-maintenance, and even reproduction, does not inherently provide reasons for humans to cherish or care about them, hence not possessing corresponding inherent worth. Again, inherent worth involves moral obligations, and since humans are the only species with the capability for moral action, the assignment of moral status is necessarily anthropocentric. When facing pathogenic microorganisms or higher-grade creatures that pose survival threats to humans, humans cannot reasonably regard maintaining their survival and development as a moral obligation.

For instance, regardless of whether viruses or bacteria are naturally occurring or artificially created, even beneficial bacteria do not obligate humans to consider their wellbeing. As long as there are justifiable reasons, we can kill or utilize them. However, for life forms higher than microorganisms, their basic biological needs, rationality, and subjective experience constitute the basic conditions for their inherent worth. Especially for potential future synthetic invertebrates, vertebrates, or even synthetic humans (again, which are likely to be ethically prohibited), the biological inherent worth is an indispensable consideration. Hence, from this perspective, considering the hierarchy of inherent objective value, the intrinsic value of synthetic life from highest to lowest is as follows: synthetic humans (if feasible), synthetic vertebrates, synthetic invertebrates, synthetic plants, and synthetic microorganisms.

### 5.2 Inherent worth from the perspective of subjectivity

The existence of subjectivity provides something that possesses it with moral reasons that humans should consider and respect. Thus, it has moral status and requires human respect and care, granting it inherent worth. But what constitutes subjectivity? How do we determine whether synthetic life has subjectivity? We believe subjectivity originates from the mind and exists in individual consciousness, involving sensation, perception, awareness, and rationality (Perry, 2009).

Firstly, sensation and perception refer to the ability to experience pleasure and pain, a highly influential standard for judging whether a life form has moral status (Sun, 2019). Positive and negative experiences, such as enjoying pleasure and avoiding pain, are considered forms of wellbeing. Sentient beings mind experiencing pain because it harms them, making pain intrinsically bad; pleasure, as a beneficial psychological experience, is intrinsically good. This ability grants life who owns it a unique moral status, forming a moral common sense that no sentient being should be killed or suffer without sufficient reason. Accordingly, humans owe them certain obligations: either to leave them alone or interact with them without cruelty.

The capacity for pleasure and pain is not unique to humans. Ignoring the suffering of animals is ethically untenable. To determine whether non-human creatures have this ability, Mary Anne Warren proposed four criteria: (1) whether the creature has a nervous system, and if so, comparing its structure and function to the human nervous system; (2) observing whether the creature exhibits behaviors such as crying, struggling, or fleeing when threatened or harmed; (3) whether it has sensory organs and exhibits behaviors indicating sensory ability; (4) whether the creature’s body contains neurotransmitters related to human experiences of pleasure or pain (Warren, 2000a). Scientific consensus indicates that vertebrates and some invertebrates possess the ability to experience pleasure and pain (Pollo and Vitale, 2019). However, even if invertebrates can feel pleasure and pain, their experiences are less intense than those of humans and other higher vertebrates. This can be inferred from their sensory organ and everyday behaviors. In contrast, microorganisms represented by viruses and bacteria almost certainly lack sensory abilities, as they possess neither sensory organs nor nervous systems, and their behaviors do not indicate any ability to experience pleasure or pain. Therefore, from this perspective, higher-grade life has greater moral status and deserves more human respect. We believe the hierarchy from highest to lowest is humans, vertebrates, invertebrates, plants, and microorganisms.

Therefore, this applies equally to synthetic life. If moral status is judged solely based on the ability to experience pleasure and pain, synthetic life at the same grade as natural life is equivalent. A typical example is transgenic salmon (though this may be controversial, we will consider it a type of synthetic life here) and naturally evolved salmon (Stevens, 2019). Since they have the same nervous systems, their sensory abilities are identical, and thus, in this sense, they have the same moral status. However, a special situation may arise, such as in the process of creating synthetic life, where the nervous system’s capacity for experiencing pleasure or pain is enhanced or diminished. A scientific example is the comparison between a standard model organism, *Caenorhabditis elegans*, and a genetically modified *Caenorhabditis elegans* with an enhanced nervous system (Fischer et al., 2022). Which has a higher moral status? As previously stated, despite differing capacities for experience, as members of the same category, we believe that their moral status should be the same.

Another important marker of subjectivity is awareness. Having sensory abilities does not necessarily mean having awareness, let alone self-awareness. Sensory reactions to pleasure and pain might merely be physiological reflexes without conscious brain processing. Compared to life forms with only sensory abilities, those with awareness and self-awareness appear more advanced and have higher moral status. Higher mammals, capable of demonstrating awareness and self-awareness, thus have a higher moral status than normal vertebrates. When facing life forms with only sensory abilities and those with awareness and self-awareness, humans should show more respect to the latter. When their interests and wellbeing conflict, humans should prioritize the latter.

However, it is difficult to determine whether a life form has awareness or self-awareness or find a clear boundary distinguishing them. Some theories link awareness with cognitive abilities and intelligence, using these cognitive manifestations as criteria for determining awareness or self-awareness (Burghardt, 2009). This view has limitations; if intelligence implies awareness and moral status, infants and individuals with intellectual disabilities would be excluded. Therefore, introducing the concept of “potential” might resolve this issue. Infants and individuals with intellectual disabilities, though not fully developed, exist in a potential state and thus possess moral status (which is why we believe moral status cannot be further divided within the same category). Thus, for synthetic life with certain or potential cognitive abilities (if realized in the future), we would consider them to have inherent worth due to their (potential) consciousness and self-awareness. As a result, they would have higher inherent worth than synthetic life with only sensory abilities, necessitating greater human respect and protection.

Finally, rationality is the most evident marker of subjectivity, currently unique to humans. There is a close conceptual link between rationality and complete moral status. Understanding it commonly uses Kant’s concept of rational agents: “Rational nature exists as an end in its … so act that you use humanity, whether in your own person or in the person of any other, always at the same time as an end, never merely as a means” (Kant, 1998a). Viewing humans as ends themselves implies seeing them as possessing “dignity” or “intrinsic value,” and our actions should align with promoting their happiness and flourishing. This can be understood on two levels: first, the obligation not to treat them merely as means, an “absolute duty” binding at all times; second, taking benevolent, beneficial actions, though not always practical, existing as an “imperfect duty.” According to Kant, rationality exists *a priori*, not derived from any empirical facts, hence applying the principle of rationality as an end itself to all rational beings (Kant, 1998b). Therefore, if, in the future, creating synthetic humans with rational capabilities and personhood becomes technologically and ethically permissible, they would possess the same inherent worth and be regarded with equal moral status as ordinary humans.

However, the notion of rationality must be understood in a nuanced manner. For example, infants and intellectually impaired individuals do not possess or are not yet capable of rationality, making the notion of “absolute duty” seem untenable, implying we have no “absolute duty” towards these groups, allowing harm, which is absolutely unacceptable. Thus, it is necessary to include groups lacking these actual capabilities but possessing potential and attributes in the scope of care. John Rawls describes moral persons as “rational beings with their own ends and capable of a sense of justice” (Rawls, 1971). It means that the minimum requirement for a moral person refers to a capability rather than the actualization of this capability. Even if these groups (e.g., infants, minors, and persons with intellectual disabilities, etc.) do not fully possess rational capabilities, having these attributes to some extent suffices to regard them as having equal moral status. Suppose the capability is the basis of inherent worth; an artificially created rational agent and a naturally formed one share the same inherent worth. Therefore, if future technology and ethics permit, not only synthetic humans but also synthetic embryos and all developmental stages of synthetic humans would possess this moral status.

In short, from the perspective of subjectivity, it is evident that (synthetic) microorganisms, (synthetic) plants, and most (synthetic) invertebrates lack moral status, while (synthetic) vertebrates have lower moral status than synthetic humans (if permitted).

### 5.3 Inherent worth from the perspective of relationships with human beings

Since the moral status of (synthetic) life is conferred by humans, in addition to considering the inherent worth of synthetic life from their own, we should also examine the inherent worth of synthetic life from the perspective of their relationships with human beings. Notably, this view is widely held by feminist ethicists and care ethicists, who believe that moral obligations cannot be understood without considering human intuition and emotions and argue that natural caring is the source of human morality. They advocate for the relevance of interpersonal, social, and emotional relationships to moral status, emphasizing the central role of human emotions in ethical theory (Noddings, 1984a). The desire to establish caring relationships is the fundamental and enduring basis of all human morality. In caring relationships, the “carer” can empathize with the feelings and needs of the “cared-for” and spontaneously meet these feelings and needs as an instinct rather than through rationality (Noddings, 1984b). In these natural caring relationships, people spontaneously exhibit different emotional care toward different lives, resulting in varying moral obligations. Nel Noddings uses the analogy of concentric circles and chains to represent these caring relationships, where the closest relationships (chains) to the center, based on love, generate the strongest obligations, and the degree of caring for different lives decreases from the inner to the outer circles, establishing a broader community through these circles and chains (Noddings, 1984c). It is important to note that this emotional relationship-based hierarchy is not targeted at an individual life, such as one’s own pets, but rather at the general emotional connection that humans have toward certain category of (synthetic) life, especially those formed through natural evolution and the sympathy or empathy arising from species similarity.

To determine the similarity of these “emotional relationships” and establish the hierarchy of moral status, one criterion is the biosocial theory, using kinship as a basis for judging the distance in the concentric circles (Warren, 2000b). Another is to judge the position in the concentric circles based on their “rational nature.” As Allen Wood suggests, the degree of moral consideration might be divided according to the similarity to human rational capabilities, including “having rational nature only potentially, or virtually, or having had it in the past, or having parts of it or necessary conditions of it” (Wood, 1998). The third criterion to assess the relationships with humans is based on subjective experience, where (synthetic) life capable of exercising the same type of agency in response to perceived subjective experiences, such as pain, share the same moral status (Sebo, 2017).

Whether based on “kinship,” “rational nature,” or “similarity in agency,” these criteria generate moral attitudes and obligations through the sympathy or empathy arising from human similarity to the species. Hence, the more similar synthetic life is to humans in these respects, or the closer its position in the concentric circles, the stronger the human moral obligations and the higher its inherent worth. From this, synthetic microorganisms lack inherent worth due to their lack of rationality, subjective experience, and extreme genetic distance from humans. By judging the hierarchical relationships conferred by these concentric circles, we conclude that when a lower-grade synthetic life conflicts with a higher-grade synthetic life, the lower-grade synthetic life should yield to the higher-grade one. For example, if creating a synthetic virus poses a threat to the health of animals, plants, or humans, such a virus should not be created.

**FIGURE 1:**
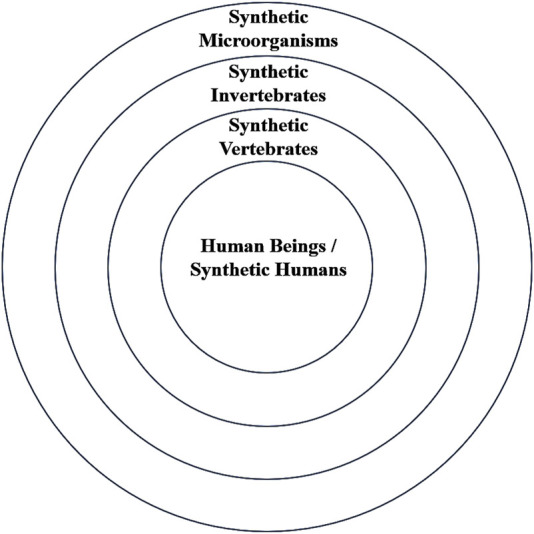
Relationship between humans and different types of synthetic life.

Thus, combining the three criteria, we can create a series of concentric circles between different types of synthetic life and humans (Figure 1). From the inner circle outward, they are synthetic humans, synthetic vertebrates, synthetic invertebrates, synthetic plants, and synthetic microorganisms, with inherent worth decreasing in that order and human moral obligations following the same descending order.

As inherent worth decreases, human moral obligations toward that type of synthetic life also decrease, making its development, utilization, and creation more justifiable. Conversely, as the “relationship” of a synthetic life to humans becomes closer, its moral status increases and human obligations toward it strengthen, weakening the legitimacy of its use. For synthetic microorganisms, humans can utilize them based on their needs, even designing “kill-switches” to address potential threats to humans and the environment. Synthetic plants and animals with sensory and perceptual abilities (potentially in the future), especially animals, should receive more care and be treated humanely without arbitrary slaughter or torture. Synthetic humans (hypothetically allowed ethically and legally) should be treated with the same moral obligations as natural humans. Specifically, creating humans involves not only the welfare and benefits of life themselves but also complex social issues, requiring careful consideration and strict prohibition under current circumstances.

## 6 Conclusion and outlook

Synthetic life possesses intrinsic value from the moment it is created. This intrinsic value can be considered from three aspects: intrinsic subjective value, intrinsic objective value, and inherent worth. However, the intrinsic value of synthetic life is not uniform and varies across different types and grades:

Intrinsic subjective value: The cultural, social, historical, and aesthetic significance of synthetic life depends on the degree to which it is valued by humans. Therefore, different synthetic lives have varying intrinsic subjective values.

Intrinsic objective value: This value is independent of the synthetic life’s mode of emergence. The natural purposes of life dictate that even fully artificially synthesized life, lacking an evolutionary history, still possesses intrinsic objective value. However, this intrinsic objective value does not provide a reason for humans not to utilize them. The derived inherent worth, which forms the basis of human moral obligations, considers not harming the synthetic life and protecting its interests.

Inherent worth: This value is examined from the perspectives of biology, subjectivity, and the relationships with human beings. Different categories of synthetic life have different moral statuses, but the moral status within the same category is equal, and the corresponding inherent worth is also the same. However, inherent worth is only present in synthetic life above the microorganisms. Neither the currently attempted “bottom-up” synthesis of “protocells” nor the “top-down” deeply modified bioengineered microorganisms possess inherent worth. Inherent worth involves not only the interests or benefits of life itself but also the moral subject’s obligation to care about their interests or benefits. Only synthetic life above the microorganisms has the basis for such moral consideration. As a result, lower-grade synthetic life, represented by protocells and bioengineered microorganisms, do not possess inherent worth, and humans have no obligations toward them. Therefore, constructing and utilizing them does not violate the overall dignity of life. Consequently, their application in fields such as medicine, environment, and energy can be justified by their significant and irreplaceable extrinsic value.

By analyzing the intrinsic value of different types of synthetic life, we aim to provide the rationale for whether synthetic life should be created and how it should be created. The discussion of the intrinsic value of synthetic life ultimately needs to be applied in governance practice. For the actions of designing, manipulating, and utilizing synthetic life, legal regulations should be established regardless of the type of synthetic life involved. Clearly, synthetic microorganisms do not have legal rights as they do not possess any moral status or inherent worth, and humans have no obligations toward them. Additionally, their intrinsic subjective and objective values are much smaller compared to humans. Therefore, in situations where human needs and wellbeing are at stake, synthetic microorganisms can be created, utilized, and even sacrificed. However, considering the important role microorganisms play in ecosystems, humans should carefully consider the biosafety and biosecurity risks involved in designing, manipulating, and utilizing them while maximizing their benefits to humanity. Synthetic plants and synthetic invertebrates have lower intrinsic subjective and objective value, as well as inherent worth, than humans. They can also be created, utilized, and sacrificed when facing human needs. However, as their intrinsic values increase, the level of importance placed on them should also increase. Especially when some plants and animals exhibit certain levels of sensation and perception, they should be given the respect that life deserves, even if personhood and legal rights cannot be granted. When dealing with synthetic vertebrates capable of experiencing pain and having certain consciousness, people should recognize their higher inherent worth or moral status, and greater moral obligations toward them. Specific protective measures and legal frameworks should be established, restricting their creation and utilization within legal bounds while taking measures to avoid harm and enhance their welfare to the greatest extent possible.

## Data Availability

The original contributions presented in the study are included in the article/supplementary material, further inquiries can be directed to the corresponding author.
